# Sex Differences in Response to Treatment with Glucagon-like Peptide 1 Receptor Agonists: Opportunities for a Tailored Approach to Diabetes and Obesity Care

**DOI:** 10.3390/jpm12030454

**Published:** 2022-03-13

**Authors:** Elpiniki Rentzeperi, Stavroula Pegiou, Theocharis Koufakis, Maria Grammatiki, Kalliopi Kotsa

**Affiliations:** Division of Endocrinology and Metabolism and Diabetes Center, First Department of Internal Medicine, Medical School, Aristotle University of Thessaloniki, AHEPA University Hospital, 54636 Thessaloniki, Greece; elpir98@gmail.com (E.R.); pegioustavroula@gmail.com (S.P.); thkoyfak@hotmail.com (T.K.); grammatikimaria@gmail.com (M.G.)

**Keywords:** GLP-1 receptor agonists, sex/gender differences, weight loss, diabetes

## Abstract

The available data suggest differences in the course of type 2 diabetes mellitus (T2DM) between men and women, influenced by the distinguishing features of the sex. Glucagon-like peptide 1 receptor agonists (GLP-1 RAs) are a relatively new class of antidiabetic drugs that act by mimicking the function of endogenous glucagon-like peptide 1. They constitute valuable agents for the management of T2DM as, in addition to exerting a strong hypoglycemic action, they present cardiorenal protective properties, promote weight loss, and have a good safety profile, particularly with respect to the risk of hypoglycemia. Due to the precedent of studies having identified sexual dimorphic elements regarding the action of other antidiabetic agents, ongoing research has attempted to examine whether this is also the case for GLP-1 RAs. Until now, sex differences have been observed in the impact of GLP1-RAs on glycemic control, weight reduction, and frequency of adverse events. On the contrary, the question of whether these drugs differentially affect the two sexes with respect to cardiovascular risk and incidence of major adverse cardiovascular events remains under investigation. Knowledge of the potential sex-specific effects of these medications is extremely useful for the implementation of individualized therapeutic plans in the treatment of T2DM. This narrative review aims to present the available data regarding the sex-specific action of GLP-1 RAs as well as to discuss the potential pathophysiologic mechanisms explaining these dissimilarities.

## 1. Introduction

Glucagon-like peptide 1 receptor agonists (GLP-1 RAs) are a relatively new class of anti-diabetic medications that have exhibited very promising results in the treatment of type 2 diabetes mellitus (T2DM) [1]. According to the 2021 American Diabetes Association guidelines, they constitute one of the preferred add-on agents when metformin monotherapy and lifestyle modifications have failed to achieve adequate glycemic control [2]. GLP-1 RAs might be useful for the treatment of people with T2DM and overweight/obesity, since they have been shown to be beneficial in achieving weight loss targets, an essential component of the therapeutic strategy of T2DM [3]. Moreover, liraglutide and semaglutide have been licensed for the management of overweight and obesity regardless of diabetes status at a dose higher than that used to treat hyperglycemia. They are also strongly recommended for the treatment of individuals with T2DM and established atherosclerotic disease or multiple cardiovascular disease (CVD) risk factors [4], due to their ability to effectively lower the risk of CVD, through various mechanisms, including antiatherogenic properties and optimal effects on blood pressure and lipid profile [5,6]. As reported by the results of a recent meta-analysis certain GLP-1 RAs, namely liraglutide and exenatide, are also considered safe and effective for the treatment of pediatric T2DM. In this study, it was mentioned that the administration of these drugs in children with confirmed insulin resistance resulted in reduction in body weight and HbA1c values. Cardiometabolic parameters did not show any significant improvement, with the exception of a slight decrease in systolic blood pressure. The main adverse effect reported after administration of the aforementioned GLP-1 RAs in the pediatric population was nausea (risk ratio 2.11) [7]. GLP-1 RAs have also been used in the treatment of children with prediabetes and/or obesity. According to another systematic review and meta-analysis, GLP-1 RAs were more effective in lowering the glycated hemoglobin (HbA1c) values in children with diabetes and prediabetes compared with children with obesity (−0.72% in children with (pre-)diabetes versus −0.08% in children with obesity). The exact opposite was demonstrated regarding the effectiveness in weight loss (−2.74 kg in children with obesity versus −0.97 kg in children with (pre-)diabetes) [8]. In accordance with the above, it appears that GLP-1 RAs act via influencing several key pathophysiologic aspects of T2DM, such as increased insulin resistance and adiposity [6]. Interestingly, both entities seem to be strongly influenced by sex hormones [9,10]. Besides, T2DM is characterized to a great extent by sexual dimorphism, which affects the presentation, diagnosis, and progression of the disease, as well as influences its potential complications. For instance, it has been shown that men with diabetes are diagnosed at an earlier age and at a lower body mass index (BMI) compared to women. On the other hand, females with diabetes present with greater levels of obesity than men with diabetes, even though males in the general population account for the majority of individuals with overweight/obesity [11]. However, differences in adipose tissue distribution should also be taken into consideration. More specifically, 70% of women with T2D present with abdominal obesity, whereas the corresponding percentage for men with T2D is approximately 40% [12]. Hence, a very strong association is indicated between T2DM and obesity in women, especially regarding the abdominal type of obesity. Similar to the above, the expected male predominance regarding CVD risk is inverted in people with diabetes, with females exhibiting higher CVD risk, possibly due to a hyperglycemia-induced loss of estrogen’s protective effect [11]. Women with T2DM present with a three- to sixfold increase in the risk of CVD compared to women without diabetes. In comparison, as far as men with diabetes are concerned, a two- to fourfold increase has been noted. The proposed risk factors for CVD may also differ between sexes. It has been recognized that increased total cholesterol and LDL levels act as significant risk factors for CVD in males, whereas in females hypertriglyceridemia and low HDL levels may constitute stronger risk factors [13]. Additional differences between sexes exist with regard to T2DM laboratory findings. It has been shown that fasting plasma glucose is more sensitive in diagnosing T2DM in males, while females exhibit a greater impairment in glucose tolerance [14]. This specific dissimilarity between sexes is extremely important since it may impact the diagnostic process of the disease. Furthermore, the prognosis of T2DM is also affected by sex. Cumulative evidence suggests that females with T2DM exhibit inferior glycemic control and are less likely to achieve their HbA1c targets [15]. They also face a higher all-cause mortality and a higher CVD-related morbidity and mortality [16].

The pathophysiology of these sex-specific differences is to a great extent hormonally regulated, with estrogen playing a key role in the disease process in females [13]. Estrogen seems to exert a protective effect on glucose metabolism in pre-menopausal women, provided that its concentration ranges within a physiological window [17]. Consequently, the post-menopausal lack of estrogen contributes to the pathogenesis of insulin resistance and T2DM. Interestingly, a hyperestrogenic environment may also lead to insulin resistance [18]. Gestational diabetes for instance serves as a great example of insulin resistance induced by the hormonal changes of pregnancy, such as elevated estrogen levels, as well as other placenta-derived hormones. In a normal pregnancy, the pancreatic β-cells undergo hypertrophy and hyperplasia. However, in an individual with pre-existing β-cell dysfunction this process of adaptation is not possible and therefore a hyperglycemic state occurs [19]. Insulin resistance associated with gestational diabetes is either transient and disappears after delivery, when hormonal levels are back to their pre-gestational state or remains impaired and leads to increased risk of T2DM in the future [20]. Apart from estrogen, testosterone levels also affect the two genders differently with regard to glucose metabolism and incidence of dysglycemia. More specifically, it has been argued that testosterone deficiency may be responsible for insulin resistance in males, whereas in females the latter can occur as a result of a hyperandrogenic state [14]. Thus, taking into consideration that T2DM is a highly sexual-dimorphic entity and that GLP-1 RAs exhibit their actions via modulating processes that are characterized by hormonal regulation, the question of whether biological sex could differentiate the response to GLP-1RAs treatment is raised. Due to the relatively recent authorization for clinical use for the treatment of T2DM, the sex-specific properties of these agents have not been adequately investigated [21,22]. The aim of this narrative review is to examine the impact of sex on the efficacy and safety profile of GLP-1 RAs and to provide valuable information that would allow targeted treatment to those who are likely to benefit the most.

## 2. GLP-1 RAs: An Overview of the Class

GLP-1 is a peptide hormone that is mostly secreted by the endocrine cells of the small intestine in response to nutrient load. Its main functions are to stimulate insulin and inhibit glucagon secretion. It also induces satiety by delaying the rate of gastric emptying and decreasing gastrointestinal (GI) motility. In addition, it has been shown that GLP-1 may play a role in the modification of gastric volume in expectation of or in response to a meal [23]. The currently available GLP-1 RAs, which act by mimicking the actions of endogenous GLP-1, are exenatide, liraglutide, albiglutide, lixisenatide, dulaglutide, and semaglutide. With the exception of oral semaglutide, all of the above agents are administered by subcutaneous injection. Due to their glucose-dependent mechanism of action, meaning that their effects are elicited only when glucose levels are elevated, GLP-1 RA therapy involves a low risk of hypoglycemia [24]. Nevertheless, possible side effects do exist, and are mainly manifested throughout the GI system consisting of, but not restricted to, nausea, vomiting and diarrhea [25]. Table 1 summarizes the characteristics of the drugs mentioned above.

## 3. Sex-Specific Efficacy of GLP-1 RAs

### 3.1. Hypoglycemic Efficacy

The pleiotropic actions of GLP-1 RAs have been in the spotlight of medical research during the past few years. However, achieving optimal glycemic control remains the primary goal of the treatment of Τ2DM, as HbA1c levels < 7% (<53 mmol/mol) are associated with a lower risk of long-term disease complications [49].

According to a large-scale retrospective pool analysis of patients receiving exenatide twice daily, the reduction in HbA1c appeared to be irrespective of sex, but highly dependent on baseline HbA1c values [21]. Similar results have been demonstrated in another pooled analysis of clinical trials that examined the use of dulaglutide in people with diabetes, in which sex did not influence the dulaglutide-mediated reduction in HbA1c level (for reference, the observed reduction in HbA1c was −1.26% in men vs. −1.33% in women) [50]. This is in agreement with a post hoc analysis of 855 patients undergoing dulaglutide treatment, in which the reduction in HbA1c was once again shown to be unaffected by sex [51].

On the contrary, several studies demonstrate a sex-specific response to GLP-1 RA with regard to HbA1c levels. A very interesting example is a study conducted in newly diagnosed T2DM individuals who were overweight or obese, without prescription of weight loss medication in the last three months and without concomitant use of oral glucose lowering agents. The results showed that women exhibited a greater decrease in HbA1c levels compared to men after the administration of combination therapy consisting of exenatide plus metformin (HbA1c levels after treatment reduced from 8.8% (73 mmol/mol) to 6.8% (51mmol/mol) in females and from 8.9% (74 mmol/mol) to 7.5% (58 mmol/mol) in males, respectively, *p* < 0.05) [6]. Additional benefits of combined treatment, with respect to adiponectin levels (which serves as an index of insulin sensitivity) [52], β-cell function and inflammation were also more pronounced in women, further supporting the concept of sexual dimorphism in response to the exenatide-metformin regimen [6]. Furthermore, a retrospective study focusing on the long-term effectiveness of exenatide indicated male sex as a predictor of treatment failure (defined as “insufficient improvement or deterioration of glycemic balance”, which corresponds to “HbA1c values > 7.5% (>58 mmol/mol) or a decrease less than 1% after one year of treatment”) with a calculated odds ratio (OR) of 2.55 [53]. Similar conclusions were drawn when examining the effects of metformin and liraglutide combination therapy. In a subgroup analysis comparing sexes, a significantly stronger reduction in HbA1c levels was noted in females compared to males (−1.5 vs. −0.75), indicating that female gender is a predictor of a better glycemic response (*p* = 0.028). However, it may be important to note that the population of this study was reported by the authors to be lacking genetic diversity, which could potentially affect the generalizability of its conclusions [5]. In another analysis investigating the efficacy of liraglutide in reducing Hb1Ac, females were more likely to achieve good glycemic control at follow-up in comparison to males (OR 1.75). Specifically, when stratified by age, female superiority was identified in the 18–64 age group, while in the over 65 age group, male predominance was observed [54]. It is plausible that these differences could be attributed to age-dependent hormonal alterations, manifested primarily as lower estrogen levels in women after menopause [55].

Other studies, however, are suggestive of a better response of men to GLP-1 RA treatment. A cohort study used exenatide twice daily as an additional agent in patients who experienced failure in metformin treatment. After 12 months of treatment, a higher percentage of male subjects achieved HbA1c target (≤7% or 53 mmol/mL) compared to females (38% vs. 27%, *p* = 0.03). Another interesting finding of this study was that the predictors of the achievement of the annual glycemic targets differed between the sexes. For males, lower baseline HbA1c levels were associated with increased likelihood of accomplishing glycemic control, whereas for females, history of previous management exclusively with metformin monotherapy was linked with lower probability of treatment failure (OR 0.321) [22].

### 3.2. Weight Loss

As mentioned above, weight loss is one of the main collateral benefits of GLP-1 RA treatment [23]. Cumulative evidence suggests that weight reduction due to GLP-1 RAs is more pronounced in females. This disproportionate response of women was evident in a retrospective study in which women exhibited on average greater weight loss compared to men (−7.0 kg vs. −3.3 kg, *p* < 0.06) [56]. Similar results were presented in another cohort study in which after a 12-month period of exenatide treatment, 33% of women achieved weight loss targets compared to 17% of men [22]. An additional study showed that the same pattern applied to BMI values, with females exhibiting greater BMI reduction at the end of the treatment period (BMI reduction: 4.8 kg/m^2^ in females vs. 2.6 kg/m^2^ in males, *p* < 0.05) [6]. Studies that used different representatives of the class, such as dulaglutide and liraglutide, have generated similar findings [50,57]. Nevertheless, the different GLP-1 RAs do not possess identical degrees of efficacy as far as weight reduction is concerned. In more detail, in a comparative study of dulaglutide versus liraglutide, the former seemed to provoke a more prominent weight loss effect. Still, in all groups examined, females consistently benefited from greater weight loss when compared with males [−1.32 kg in females (*p* = 0.001) vs. + 0.09 kg in males for dulaglutide (*p* < 0.001) and −0.51kg (*p* = 0.413) vs.−0.03 kg (*p* = 0.014) for liraglutide, respectively)] [57].

The mechanism behind the better female response to weight reduction remains unclear, but it could potentially be associated with increased drug exposure observed in women, possibly due to their lower average body weight [57]. Interestingly enough, another study with liraglutide produced opposing results, as it concluded that in this case, in addition to female sex, a higher baseline BMI was also associated with greater weight loss. It should be noted that in this particular case, the female participants were slightly heavier than the males at the beginning of the study [58]. To these conflicting results, an explanation could be potentially provided by examining the findings of a pharmacokinetic analysis on liraglutide. This analysis showed that liraglutide exposure was 32% higher in women than in men of comparable weight, thus identifying the female sex as an independent predictor of weight loss achievement [59]. 

### 3.3. Cardiovascular Risk and Major Adverse Cardiovascular Events (MACE) 

Aside from their previously mentioned benefits, GLP-1 RAs exert several other favorable effects such as the decrease of waist circumference (WC) [60] and blood pressure (BP) [61], as well as the modification of various elements of the lipid profile, thus resulting in reduced CVD risk [62]. This is of utmost importance, since it is well established that patients with T2DM are characterized by a high risk of CVD, which is the main cause of morbidity and mortality in this population. In this concept, it is essential to examine whether the effects of GLP-1 RAs on certain variables that serve as recognized modifiable regulators of CVD risk exhibit sexual dimorphic patterns [63].

With respect to the above, a study of 179 patients receiving liraglutide treatment concluded that even though the reduction in HbA1C was more evident in women, WC and BP decreased significantly in both sexes to a similar extent [5]. The absence of sex-related differences in the control of BP levels was also supported by a pool analysis of exenatide twice daily [21]. However, according to this analysis, low-density lipoprotein cholesterol (LDL-C) did not decrease in women, indicating the presence of a specific sex effect of exenatide on lipid profiles [21]. However, even though several studies have explored the link between GLP-1 RAs and parameters that influence CVD risk, only a small number of them have stratified their data by sex. 

Approaching this matter from a different perspective, the sex specificity of GLP-1 RAs on CVD risk could also be examined by measuring the incidence rates of MACE. Although the definition of MACE might vary between different trials, it generally encompasses the major complications of CVD that correspond to nonfatal myocardial infarction, nonfatal stroke, and cardiac death, among others [64].

Only a few studies examined the potential sex-specific protective effect of GLP-1 RAs on MACE, occurring as a diabetic complication [65]. This is of great interest because the population of patients with diabetes manifests a distinct epidemiological composition; as already mentioned above, in contrast to the male predominance observed in the general population as far as cardiovascular events are concerned, in diabetic individuals this risk appears to be higher in females [66].

Studies that examined the efficacy of GLP-1 RA with respect to cardiovascular outcomes noted a remarkable reduction in MACE in both sexes [67,68], although without apparent sex-related divergence (hazard ratio of MACE occurrence 0.88 in both sexes) [68]. This was further supported by a meta-analysis that concluded that the effect of GLP-1 RAs on MACE was similar between men and women (*p* = 0.375) [69]. A conflicting conclusion was drawn by another study which demonstrated that the use of GLP-1 RAs was linked to a lower frequency of MACE in women than in men (incidence rate: 6.6 per 1000 person-year [PY] for females versus 11.9 per 1000 PY for males, *p* < 0.001). Furthermore, when comparing sulfonylureas with GLP-1 RAs, the risk of MACE was found to be lower in those treated with the latter. An important aspect of this comparative study is that the reduction in risk associated with GLP-1 RA treatment was even higher in women than in men (adjusted hazard ratio in patients treated with GLP-1 RA vs. patients treated with sulfonylurea was 0.57 in females vs. 0.82 in males, *p* = 0.001), further suggesting a two-way drug-by-sex interaction [70]. 

## 4. Sex as a Determinant of Different Adverse Outcomes of GLP-1 RA Treatment

In general, GLP-1 RAs possess a good safety profile with minor adverse events. The most commonly reported side effects of these drugs are GI adverse events (GI AE) such as nausea and vomiting, while symptoms that are less frequently encountered are diarrhea and constipation. Additionally, there have been a few associations with incidents of major adverse outcomes such as episodes of acute pancreatitis and thyroid malignancy. However, these have been observed mainly in animal studies and a cause–effect relationship among GLP-1 Ras, and these outcomes have not been established in human clinical trials [71].

In an analysis of data derived from two randomized control trials, testing monotherapy with dulaglutide versus liraglutide for the treatment of T2DM, more women reported overall treatment-emergent adverse events compared to men in both arms of the study (86.5% of women vs. 61.4% of men for dulaglutide and 83.3% vs. 65.5% for liraglutide, respectively). GI AE in particular also occurred more frequently in women. Interestingly, patients with higher percentages of GI AE were more likely to achieve greater weight loss. Thus, sex-related differences in GI AE occurrence may be indirectly associated with increased GLP-1 RA efficacy among women.

The apparent sex-specific differences in the frequency of GI AE and in the efficacy of GLP-1 RA treatment were partially attributed by the authors of this analysis to higher exposures (defined by the authors as “higher drug plasma concentrations”) of dulaglutide and liraglutide in females, possibly due to their lower average body weight [57]. The association between higher levels of GLP-1 RA exposure and a higher frequency of adverse GI events has already been highlighted in several studies. For instance an exposure-focused study of liraglutide identified nausea and vomiting as established exposure-dependent adverse events of liraglutide treatment [72]. Nonetheless, the findings of an exposure–response analysis of semaglutide provided more insight into the matter by concluding that these particular GI AE, although being exposure dependent in general, were more frequent in females across similar levels of exposure [73]. This could potentially suggest that exposure does not comprise the main determinant of the sex-differences observed in the incidence of GI AE after treatment with GLP-1 RAs. Table 2 provides a more detailed view on the relationship between exposure/dose, effectiveness and AE of GLP-1 RAs.

**Figure 1:**
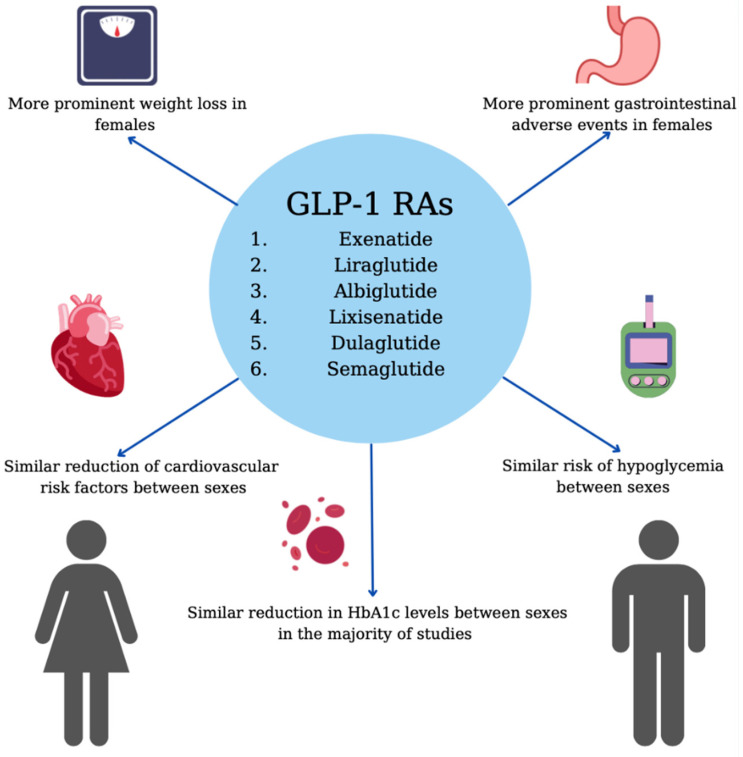
Sex-specific effects of GLP-1 RAs.

Further exploring this subject, a study analyzing data derived from two national Korean databases concluded that females were approximately twice as likely than males to report any adverse events linked to GLP-1 RA use, with a reporting ratio of 2.34 (reporting ratio: reporting rate of women/reporting rate of men). Regarding GI AE, the higher percentage reported by women could be partially attributed to the greater prevalence of functional GI disorders among the latter. Another interesting finding of this analysis was that women had a higher probability of experiencing headaches after treatment with GLP-1 RAs compared to men (risk ratio of 7.97) [76]. In contrast to the above, the percentage of patients having experienced episodes of nocturnal or total hypoglycemia did not differ significantly between sexes [57]. Figure 1 describes the sex-specific effects of GLP-1 RAs.

## 5. Discussion

It is beyond doubt that GLP-1 RAs constitute an effective tool in the management of T2DM and obesity. However, the hypothesis of whether sex could pose as a significant factor determining the efficacy and tolerability of GLP-1 RA treatment has been postulated and remains to be verified. In more detail, although several studies have indicated that sex may comprise a predictor of HbA1c reduction after the initiation of GLP-1 RA treatment, other studies have not found such a correlation, and therefore a definitive conclusion has not yet been drawn on the matter. In contrast, female sex appears to be a well-recognized independent factor linked to greater weight loss achievement after treatment with GLP-1 RAs. This is also the case for adverse events resulting from the use of these medications, which appear to manifest in higher percentages in women, mainly affecting the GI tract. According to existing studies, no conclusive result has emerged on whether sex may influence other parameters, such as WC, BP, lipid profile, and incidence of cardiovascular events. Table 3 summarizes the above-mentioned sex differences with respect to treatment with GLP-1 RAs.

Regarding the topic of glycemic efficacy, it is important to point out that sex-specific differences have been previously identified with respect to other antidiabetic agents. For example, insulin glargine has been found to benefit males more than females. More specifically, a pooled analysis of nine randomized control trials demonstrated that men who received insulin glargine were more likely to achieve the glycemic target (HbA1c < 7% or 53 mmol/mol) in comparison to women (percentage of achievement of glycemic control: 60.8% in males versus 54.3% in females, *p* = 0.0162). The same was noted for treatment with neutral protamine Hagedorn insulin (*p* = 0.0262) [77]. Another study examining the results of dapagliflozin treatment, a sodium glucose cotransporter 2 inhibitor, demonstrated that male sex was a predictor of a greater reduction in HbA1c (*p* < 0.05) [78]. However, not all anti-diabetic drugs exhibit sexual dimorphic properties. A case in point is metformin, the first-line treatment drug for T2DM, which produces similar results in terms of glycemic efficacy between the two sexes [79,80].

Approaching the matter of sexual dimorphism of T2DM from a different angle, current research has shed light on several anatomical and functional dissimilarities between the female and male pancreas. More specifically, according to a study conducted on biopsies of human pancreases, the percentage of β-cells in the female pancreatic islets appeared to exceed that of the male islets (6% more β-cells on average in females) [81]. In contrast, another study of human pancreatic islets from 87 human pancreases showed no statistically significant difference in β-cell number between sexes. However, this same study highlighted the presence of different patterns of methylation between male and female islets, mainly with regard to the X chromosome and especially involving various genes that control insulin secretion and function. One of these genes is the Dual Specificity Phosphatase 9 (DUSP9) gene, which is protective against insulin resistance and which exhibited a higher percentage of methylation in female pancreatic islets. Increased methylation of a gene’s promoter is known to induce gene silencing [82], and thus increased methylation of the DUSP9 gene could act as a risk factor for insulin resistance and T2DM [83]. According to recent data stemming from the use of single-cell RNA sequencing, several both sex-specific and sex-independent differentially expressed genes (DEGs) related to insulin secretion and pathophysiology of diabetes have been identified in healthy and diabetic mice. With regard to sex-specific DEGs, the insulin II gene may serve as an example since it appeared to have a more pronounced expression in male compared to female β-cells of healthy mice. In the same study, other T2D sex-specific DEGs were identified in diabetic mice in comparison with healthy mice, some showing female superiority, while others being expressed to a greater extent in male mice. In females, some of these DEGs were associated with impaired endoplasmic reticulum stress responses. In male mice, specific upregulated DEGs were associated with mitochondrial function, which is known to affect the process of insulin secretion. All of the above indicate a high level of sexual dimorphism regarding the transcriptome of β-cells in healthy and diabetic mice. These data therefore support the notion that sex plays a pivotal role in the pathophysiology of T2DM and thus may be an important factor to consider when opting for personalized T2DM management. However, the two sexes share some aspects of the disease pathogenesis since sex-independent DEGs involving several disease-specific processes were also demonstrated [84].

Taking into consideration all of the above, it is evident that some aspects of GLP-1 RA pathophysiology and mechanism of action need to be further evaluated for better guidance regarding their use in the clinical setting. More specifically, since there seems to be a more prominent effect of GLP-1 RAs regarding weight reduction in women, it would be beneficial to shed light on the exact root of such differentiation. Therefore, more research needs to be done focusing not only on the role of increased drug exposure in women, but also on the impact of the female hormonal profile on the efficacy of GLP-1 RAs. Animal studies examining the impact of sex on the anorexigenic effects of GLP-1 RAs have demonstrated that, at least in part, sex hormones are responsible for the differential responses between males and females. More specifically, it has been shown that estrogen signaling is a crucial component via which GLP-1 RAs modify the food-reward aspect of food seeking. In this sense, higher estrogen concentrations in females, especially during their premenopausal years, could potentially serve as an explanation for why women may respond better to GLP-1 RAs [85]. This hypothesis is further supported by studies exploring the fluctuations in food intake of women throughout their menstrual cycle. These studies have observed that food intake tends to be lower during the follicular phase and the periovulatory period, albeit higher during the luteal phase of the cycle, thus exhibiting an inverse correlation to estrogen levels [86]. Prompted by these findings, some researchers have suggested the combined administration of GLP-1 RA and estrogen by developing a new conjugate molecule. The goal of this newly developed drug would be to hypothetically maximize weight loss results and achieve better control of metabolic syndrome, while simultaneously limiting the potential adverse gynecological events and tumorigenesis potential of estrogen, by ensuring targeted estrogen delivery specifically to GLP-1 receptor expressing cells [87]. A very interesting element of this strategy is that when administered as a conjugate molecule, GLP-1 RAs and estrogen act in a synergistic manner, activating a specific brain area which controls food intake, the supramammillary nucleus. This activation results in a greater modification of the food-reward behavior and therefore can potentially lead to a greater reduction in body weight [88]. Nonetheless, this approach has been primarily tested in animal studies and requires further research to be introduced in the clinical setting. Should substantial evidence supporting this correlation arise, the potential of co-administering estrogen with GLP-1 RAs as single-molecule peptides should be further evaluated. 

Additionally, it is of utmost importance to clarify whether GLP-1 RA treatment exhibits sex-specific properties regarding the reduction in cardiovascular risk, by modifying several parameters such as WC, BP and lipids, as well as the decrease of MACE frequency. Should such a sexual dimorphism be demonstrated, physicians might have more opportunities to develop a tailored approach to diabetes and obesity, seeing that certain patients may benefit from earlier initiation of specific treatment.

Another aspect that should not be overlooked when attempting to examine the sexual dimorphic properties of GLP-1 RA treatment is the potential sex-specific differences encountered with regard to treatment adherence. In further detail, a study focusing on a variety of factors influencing adherence rates to noninsulin antidiabetic agents indicated that male sex was independently associated with greater treatment adherence (OR 1.14, *p* = 0.0001) [89]. These results were in accordance with the conclusions drawn by a retrospective study measuring the adherence and discontinuation rates of injectable GLP-1 RAs. At the 12- and 24-month time points after the initiation of GLP-1 RA, the percentage of men who qualified as adherent exceeded that of women (adherence rate: 54.2% for men versus 48.4% for women at 12 months and 51.1% versus 44.6% at 24 months, respectively, *p* < 0.01). With regard to the same time intervals, males also presented with lower rates of discontinuation of treatment (*p* < 0.01) [90]. These data suggest that adherence to prescribed treatment could be considered a factor that differentially affects the efficacy of GLP-1 RAs, possibly not allowing women to obtain the full benefit of their course of treatment. As a result, a sex-specific pattern in adherence rates may possibly obscure further primary differences in treatment outcomes between sexes. Hence, this subject could constitute a potential research topic in future studies, which could further clarify the impact of differences in adherence rates on the sexual dimorphism of GLP-1 RA treatment, whereas at the same time, achieve a better understanding regarding the sex-specific mechanism of action of these medications.

Our review of the literature has a number of limitations. Firstly, the majority of referenced studies are characterized by extremely heterogeneous patient populations. More specifically, the patient population in most studies differs with regard to duration of diabetes, current pharmacologic regimen, and/or previous medication history, among other factors. This lack of uniformity may confound some additional parameters that may influence the final results of each study. Furthermore, most of the available studies that examine the impact of sex on the outcome of GLP-1 RA treatment are post hoc analyses and, therefore, their conclusions constitute secondary measure outcomes. It is also important to note that although in several cases the association of GLP-1 RA with a certain variable had been evaluated, sex stratification was not conducted. Hence, the lack of sex-disaggregated data seems to be a fundamental limitation of our report and a major exclusion criterion for many other relevant studies currently available in the literature.

## 6. Conclusions

In conclusion, it is evident that the male and female responses to GLP-1 RAs differ at some level with respect to the achievement of weight loss and the incidence of GI AE. Regarding other parameters, namely HbA1c levels and cardiovascular risk, it remains to be seen whether there is a sex difference. This difference could be partly attributed to the sex specific pharmacodynamics of GLP-1 RAs which lead to dissimilar drug exposure levels. Alternatively, this dimorphism could be attributed to specific hormonal profiles of the sex. However, clinical trials do not always provide sufficient sex-specific data, which is of paramount importance to shed more light on the sex-specificity of the GLP-1 RA action. Thus, henceforward, sex-disaggregated data should stand in the epicenter of the research field, in order to enable the drawing of more accurate scientific conclusions and optimize the management of T2DM.

## Figures and Tables

**Table 1:** Characteristics of GLP-1 RAs.

	Exenatide	Liraglutide	Albiglutide	Lixisenatide	Dulaglutide	Semaglutide
Molecular weight (Dalton)	4187 [26]	3751 [27]	3283.6 [28]	4858 [29]	59,669 [30]	4114 [31]
Molecular formation	C_184_H_282_N_50_O_60_S [26]	C_172_H_265_N_43_O_51_ [27]	C_148_H_224_N_40_O_45_ [28]	C_215_H_347_N_61_O_65_S [29]	C_2646_H_4044_N_704_O_836_S_18_ [32]	C_187_H_291_N_45_O_59_ [31]
Structure	Natural peptide (exendin-4) from the saliva of the lizard Heloderma suspectum (53% homology) [33]	Slightly modified GLP-1 (97% homology) with free fatty acid side chain attached [33]	Two modified GLP-1 molecules amino-terminally attached to the linear structure of albumin [33]	Exenatide plus poly-lysine tail [33]	Two modified GLP-1 molecules attached to an immunoglobulin (Fc) fragment [33]	Slightly modified GLP-1 (94% homology) with free fatty acid side chain attached [33]
Time to peak (h/days)	2.1–2.2 h [34]	11.0–13.75 h [35]	3–5 days [36]	≈2 h [37]	48 h [38]	24 h (subcutaneous injection) [39]
Elimination half-life (t 1/2)	3.3–4 h [34]	12.6–14.3 h [35]	5.7–6.8 days [36]	2.6 h [37]	4.7–5.5 days (0.75 mg); 4.7 days (1.5 mg) [40]	7.6 days [39]
Drug-drug interactions	Drug-drug interactions with digoxin, lovastatin, lisinopril, and acetaminophen [41]	Very low potential for pharmacokinetic drug–drug interactions related to cytochrome P450. No clinically relevant interactions between steady-state liraglutide and insulin detemir, atorvastatin, griseofulvin, paracetamol, digoxin, lisinopril or oral contraceptives [42]	Coadministration with chloroquine, hydroxychloroquine, lanreotide, octreotide, pasireotide, thioctic acid is not recommended [43]	Delays gastric emptying and can reduce the rate of absorption of oral medications such as acetaminophen, ethinyl estradiol, and warfarin. Does not affect the activity of cytochrome P450 isoenzymes [44]	Delays gastric emptying and can reduce the rate of absorption of oral medications. Concomitant use with an insulin secretagogue (e.g., sulfonylurea) or with insulin may increase the risk of hypoglycemia [38]	Minor delay of gastric emptying. No clinically relevant effect on the exposure of metformin, warfarin, atorvastatin or digoxin [45]
Adverse effects	Nausea, vomiting, diarrhea, dyspepsia, dizziness, headache [41]	Nausea, vomiting, diarrhea, dyspepsia, constipation, injection site reactions, low incidence of hypoglycemia [42]	Nausea, vomiting, diarrhea, constipation, gastroesophageal reflux disease, abdominal pain [46]	Nausea, vomiting, diarrhea. Concomitant use with an additional medication known to cause hypoglycemia can increase the risk of the latter [44]	Nausea, vomiting, diarrhea, abdominal pain, decreased appetite, hypoglycemia [47]	Nausea, vomiting and diarrhea, increased risk of cholelithiasis [48]

**Table 2:** Relationship between exposure/dose, effectiveness and AE of GLP-1 RAs.

	Exposure–Response Analyses of Semaglutide (Kristin C.C. Petri et al.) [73]	Exposure–Response Analyses of Liraglutide (J.P.H.Wilding et al.) [72]	Dose-Finding Study of Semaglutide (Michael A. Nauck et al.) [74]
HbA1c reduction	Exposure dependent	Exposure dependent	Dose dependent
Body weight loss	Exposure dependent	Exposure dependent	Dose dependent
Increase in pulse rate	Exposure independent	Exposure independent (*p*-value for slope = ∼0.18)	Dose dependent
Episodes of nausea	Exposure dependent	Exposure dependent for episodes of any severity (*p*-value for slope = 0.004)	Dose dependent
		Exposure independent for moderate/severe episodes (*p*-value for slope = 0.90)	
Episodes of vomiting	Exposure dependent	Exposure dependent for episodes of any severity with doses up to 1.8 mg	Dose dependent
		Exposure independent for moderate/severe episodes (*p*-value for slope = 0.85)	
Diarrhoea	Exposure independent	Data not provided	Dose dependent
Constipation	Exposure independent	Data not provided	Data not provided
Elevated calcitonin levels (biomarker of C-cell activity and mass [75])	Exposure independent	Exposure independent (*p*-value for slope = ∼0.49)	No effect
Hypoglycemia	Data not provided	Exposure independent (*p*-value for slope = 0.83)	Dose independent
Adverse effects of the gallbladder, malignant neoplasms, malignant breast neoplasms or benign colorectal neoplasms	Data not provided	Exposure independent	Data not provided
Acute pancreatitis	Data not provided	Exposure indepndent	No effect

**Table 3:** Sex differences with respect to treatment with GLP-1 RAs. RAs: glucagon-like peptide 1 receptor agonists; CVD: cardiovascular disease; MACE: major adverse cardiovascular events; WC: waist circumference; BP: blood pressure; GI AE: gastrointestinal adverse events; LDL-C: low-density lipoprotein cholesterol.

	Differences in Response
Hypoglycemic efficacy	No sex differences noted in the majority of studies (ref. [21,50,51]) Female superiority noted in a few studies (ref. [5,6,52,53,54]) Male superiority noted in one study (ref. [22])
Weight loss	Female superiority noted in the majority of studies (ref. [6,22,50,56,57])
MACE CVD risk factors i. WC ii. BP iii. Lipid profile	No sex differences noted (ref. [68,69]) Higher risk in females (ref. [66] Lower risk in females (ref. [70]) Similar reduction between sexes (ref. [5]) Similar reduction between sexes (ref. [5,21]) Similar alterations (except for no reduction of LDL-C in females ref. [21])
Adverse events i. GI AE ii. Headaches iii. Hypoglycemia	More frequent in females (ref. [57,73,76]) More frequent in females (ref. [76]) No sex differences noted (ref. [57])

## Data Availability

Not applicable.
